# Cathepsin D SNP associated with increased risk of variant Creutzfeldt-Jakob disease

**DOI:** 10.1186/1471-2350-9-31

**Published:** 2008-04-21

**Authors:** Matthew T Bishop, Gabor G Kovacs, Pascual Sanchez-Juan, Richard SG Knight

**Affiliations:** 1 National CJD Surveillance Unit, Bryan Matthews Building, Western General Hospital, Crewe Road, Edinburgh, EH4 2XU, UK; 2 Institute of Neurology, Medical University Vienna, Vienna, Austria; 3 Institute for Formation and Research of the Fundación "Marqués de Valdecilla" (IFIMAV), Santander, Spain; 4 Centro de Investigación Biomédica en Red sobre Enfermedades Neurodegenerativas (CIBERNED)

## Abstract

**Background:**

Variant Creutzfeldt-Jakob disease (vCJD) originally resulted from the consumption of foodstuffs contaminated by bovine spongiform encephalopathy (BSE) material, with 163 confirmed cases in the UK to date. Many thousands are likely to have been exposed to dietary infection and so it is important (for surveillance, epidemic modelling, public health and understanding pathogenesis) to identify genetic factors that may affect individual susceptibility to infection. This study looked at a polymorphism in the cathepsin D gene (refSNP ID: rs17571) previously examined in Alzheimer's disease (AD).

**Methods:**

Blood samples taken from 110 vCJD patients were tested for the C-T base change, and genotype data were compared with published frequencies for a control population using multiple logistic regression.

**Results:**

There was a significant excess of the cathepsin D polymorphism TT genotype in the vCJD cohort compared to controls. The TT genotype was found to have a 9.75 fold increase in risk of vCJD compared to the CT genotype and a 10.92 fold increase compared to the CC genotype.

**Conclusion:**

This mutation event has been observed to alter the protease activity of the cathepsin D protein and has been linked to an increase in amyloid beta plaque formation in AD. vCJD neuropathology is characterised by the presence of amyloid plaques, formed from the prion protein, and therefore alterations in the amyloid processing activity of cathepsin D may affect the neuropathogenesis of this disease.

## Background

Variant CJD is one of the prion diseases, which affect both humans and animals. It is an acquired disease associated with infection with bovine spongiform encephalopathy (BSE), mainly through the ingestion route [1,2], although three cases have resulted from human-to-human blood transfusion. [3-5] It is most prevalent in the UK, where 163 confirmed cases have been reported, but has also been found in smaller numbers in other countries including France (24 cases, see Availability and requirements section for URL). It is estimated that over 500,000 BSE infected cattle entered the human food chain [6] and therefore a significant number of people may have been exposed to infection. One explanation for the current low number of cases is that there may be genetic determinants or influences on susceptibility to infection. One definite significant genetic risk factor so far identified is being homozygous for methionine (MM) at codon 129 of the prion protein gene (*PRNP*). An individual can be homozygous for methionine (MM) or valine (VV) or a heterozygote (MV), and healthy UK controls show genotype frequencies of 42% MM, 47% MV, and 11% VV. [7] All tested patients with vCJD have been *PRNP*-129 MM. Homozygous individuals are also found more frequently in patients with sporadic CJD [8] and iatrogenic CJD. [9] Attempts to find other genetic associations have used both human patient samples and experimental animal models. Analysis of the prion protein gene promoter region identified three polymorphisms that were more common in patients than controls however due to the low numbers of samples tested there was only a weak statistical association. [10] Human leukocyte antigen (HLA) typing in vCJD patients (n = 50) indicated that there was a reduction in frequency for the class-II type DQ7 compared to sporadic CJD patients [11] however this association was not maintained on testing a larger sample size. [12] Detailed analysis of a 35 kb sequence flanking the prion gene locus identified 56 single nucleotide polymorphisms (SNPs) and one of these showed strong association with sCJD but not vCJD or iatrogenic CJD. [13] Mouse models of TSE transmission can provide candidate regions for analysis in the homologous regions of the human genome. Three such studies have been published and each has suggested regions of the mouse genome that have significant association with incubation time changes following challenge with TSE agents. [14-16] These findings have yet to be assessed further in the homologous human chromosomal regions.

For this study we selected a SNP in the gene coding for the cathepsin D protein (*CTSD*), that had already been the subject of extensive publications in the field of Alzheimer's disease (AD) a neurodegenerative disease with some pathological similarities to CJD. (For meta-analysis of the association of this SNP with risk of AD see: [17]) Cathepsin D is a ubiquitously expressed lysosomal protease involved in proteolytic degradation, cell invasion, and apoptosis. Aberrant expression of the gene can lead to neurodegeneration in both experimental animal models and humans. [18] Of relevance to this study cathepsin D has been shown to exhibit a beta-secretase-like role [19], and could therefore cleave beta amyloid precursor protein (APP) and promote the cascade leading to deposition of extracellular amyloid beta-peptide (Aβ), the senile plaques found in Alzheimer's disease. Additionally, cathepsin D has also been suggested to cleave Aβ peptides and therefore clear the aggregated protein deposits. [20] Limited publications regarding cathepsin D and forms of CJD have focused on detection of the protein by immunohistochemical and morphometrical methods. On one hand these studies have shown co-localisation with the disease associated form of the prion protein [21,22]. On the other hand a significant correlation between tissue pathology and volume of neuronal cathepsin D immunoreactive lysosomes was demonstrated [21] emphasizing the role of cathepsin D in the pathogenesis of prion disease.

The gene encoding cathepsin D (*CTSD*, OMIM: 116840) is situated on the short arm of chromosome 11 (11p15.5) and consists of nine exons. On exon two there is a SNP at position 224 corresponding to a C-to-T transition resulting in a alanine to valine substitution in the translated protein (refSNP ID: rs17571, RefSeq: NM_001909, Variation: g.804C>T). Publications have indicated that this SNP may: modify the secretion or maturation of the cathepsin D profragment [23]; lead to a reduction in cerebrospinal fluid (CSF) tau concentration in Alzheimer's disease [24]; and show a decrease in general intelligence testing scores in a healthy older population [25]. The association of this SNP with risk of Alzheimer's disease and the presence of particular pathological features seems to be linked with the apolipoprotein E (APOE) genotype, but shows variability amongst populations tested. [17,26-28] A United Kingdom based study on this SNP has indicated a genetic association between the SNP, APOE genotype, and pathological features of Alzheimer's disease (Aβ deposition). [29]

This study aimed to test the *CTSD *rs17571:C>T SNP allele and genotype frequencies in a cohort of variant CJD patients. The deposition of aggregated prion protein amyloid, characteristically in the form of florid plaques, may be affected by the modified cathepsin D activity through the *CTSD *rs17571:C>T SNP, and as such may affect the risk of an individual developing vCJD.

## Methods

Blood samples for this study were taken from 110 patients during investigation of their case histories in respect to diagnosis of variant CJD. Consent was given for research and the study is covered by approval from the Lothian Health Board, Lothian Research Ethics Committee (reference MCO/103/90). Each patient was later confirmed as vCJD, with a classification of either 'definite' or 'probable' vCJD by either neuropathological examination of post mortem tissue [30] or by fulfilment of the WHO-adopted diagnostic criteria (see Availability and requirements section for URL) respectively. The median age at onset of symptoms was 27 (range 12–62) and the mean duration of the clinical phase was 15 months (range 6–39). 59% (n = 65) of the patients were male.

DNA was extracted from whole blood using the Qiagen Blood Mini Kit. A 317 bp region of exon 2 containing the *CTSD *rs17571:C>T SNP was amplified following the published protocol. [25] The forward oligo was 5'-GTGACAGGCAGGAGTTTGGT-3' and the reverse was 5'-GGGCTAAGACCTCATACTCACG-3', and the cycling program was a standard touchdown method (annealing temperature reducing from 65°C to 60°C over 10 cycles). The amplicon was digested with restriction endonuclease MwoI (NEB, UK) and products were run on a 1.5% agarose gel, visualised with ethidium bromide.

Allele frequency comparisons were made to UK control data published by Davidson et al from 767 mentally normal people aged >50 years. [29] Hardy-Weinberg equilibrium (HWE) was assessed in the control population and the patients using χ^2 ^statistics. Genotypic and allelic frequencies were compared using multiple logistic regression. All statistical analyses were performed in SPSS software (version 13).

## Results

Table 1 shows the *CTSD *rs17571:C>T SNP genotype frequencies from analysis of 110 vCJD patients, together with the published control data. *CTSD *rs17571:C>T SNP genotype frequencies were in HWE in patients (p = 0.097) and controls (p = 0.16). The mutant allele T is present in the homozygous state in a very small number of controls (0.3%, n = 2 of 767 tested) but significantly has been found in almost ten-times more vCJD patients (2.7%, n = 3 of 110 tested). Clearly the numbers of TT individuals are rare however statistical analysis (Fisher's exact test) of the SNP genotypes indicates that the differences found are of statistical significance. Odds Ratio analysis shows that compared with CT and CC, the TT genotype increases the risk of vCJD by 9.75 fold and 10.92 fold respectively. The 95% confidence intervals are wide as a result of the small numbers of TT individuals.

**Table 1:** Summary of *CTSD *rs17571:C>T SNP genotyping and statistical analysis for vCJD patients and UK controls [29].

***CTSD *rs17571: C>T SNP**	**vCJD n (%)**	**Controls n (%)**	**Fisher's exact test p-value**	**OR (95% CI); p-value**
**CC**	89 (80.9)	648 (84.5)	0.023	OR TT/CC = 10.92 (1.80–66.26); 0.009
**CT**	18 (16.4)	117 (15.3)		OR TT/CT = 9.75 (1.52–62.47); 0.016
**TT**	3 (2.7)	2 (0.3)		
**C**	196 (89.1)	1413 (92.1)	0.13	OR T/C = 1.43 (0.90–2.27); 0.11
**T**	24 (10.9)	121 (7.9)		

Statistical analysis was performed to determine whether variation in the clinical features present in vCJD patients was associated with the *CTSD *genotype. Neither the age at onset nor clinical duration of illness showed an association (ANOVA and Kruskal-Wallis tests respectively). The three vCJD patients that were homozygous TT for the SNP showed clinical and neuropathological features typical of vCJD.

## Discussion

The identification of genetic risk factors that increase an individual's chance of developing vCJD would help to understand why the present number of cases is low, despite a potentially significant population exposure to infection, and also assist in the prediction of the number of future cases. Genetic factors are likely to be those that affect expression, maturation, or function of a protein and therefore may provide evidence for cellular processes that play key roles in vCJD disease pathogenesis (which remains incompletely understood). The characterisation of prion protein and amyloid precursor protein gene mutations show direct links to familial CJD and Alzheimer's respectively, however these diseases like many others are likely to be affected by a significant number of other genomic and proteomic components. Genomic approaches to studying vCJD have yet to be published and may be difficult to interpret due to the small number of cases available for testing, so we are reliant on findings from experimental animal models and other diseases that involve neurodegeneration and protein misfolding/aggregation, such as Alzheimer's and Huntington's disease.

Currently the only polymorphic gene sequence that shows direct association with vCJD is the prion protein gene codon 129. All patients so far tested (146/163) were methionine homozygous (MM) a genotype present in only ~40% of the UK population. The idiopathic form of CJD (sporadic CJD) similarly shows an over-abundance of MM in patients (~70%) [8] and therefore this genotype has a significant risk associated with development of these human prion diseases. It is proposed that all codon 129 genotypes may be susceptible to vCJD and that the MM hosts have the shortest incubation time. [31-33] MV and VV individuals may present with vCJD in second and third waves of the epidemic at a later date. [34] It is also possible that these few specific MM individuals have additional genomic variations that have significant additive effects, leaving them highly susceptible to developing vCJD.

This study aimed to assess the influence of a missense polymorphism (C-T; Alanine – Valine) at position 224 of the cathepsin D protein on risk of vCJD. This polymorphism has been extensively investigated with respect to Alzheimer's disease (AD). A meta-analysis from 2004 [17] covering 14 studies concluded that *CTSD *rs17571:C>T was not a major risk factor for AD, however in conjunction with the APOE4 genotype it had an effect on susceptibility. There was significant between-study heterogeneity possibly due to comparing data from many countries with variable *CTSD *rs17571:C>T genotype frequencies. The vCJD samples available for this study were from Caucasian patients resident in the UK. The control group were also Caucasian individuals from the UK. It is therefore believed that geographical origin of patients and controls would not affect the analysis.

A recent UK study, from which we have taken the control population frequencies, found a correlation between *CTSD *rs17571:C>T and the pathogenesis of AD. [29] Specifically there was an increase in the number of senile plaques containing Aβ_40 _in the brain associated with the T allelic variant of cathepsin D. This variant is thought to have a greater beta-secretase activity than the C variant therefore yielding more of the cleaved Aβ which in turn may accelerate AD pathogenesis by seeding amyloid formation [23,29,35]. Risk of developing AD was not found to be directly affected by the possession of the *CTSD *rs17571:C>T T allele.

There remains a possibility that the *CTSD *rs17571:C>T SNP is in linkage disequilibrium with another neighbouring gene on chromosome 11 that may be the direct cause of the findings in this study. *CTSD *is located in a region of chromosome 11 (11p15.5) that contains genes for a number of transmembrane proteins and cell surface glycoproteins, such as CD81, SYT8, TSPAN4, TSPAN32, that may have roles in common with prion protein. Also present is DUSP8, a phosphatase inhibitor of members of the mitogen-activated protein kinase (MAPK) superfamily that are associated with cell differentiation, proliferation, and apoptosis in the adult brain.

## Conclusion

The study detailed here found an over-representation of the *CTSD *rs17571:C>T T allele in UK vCJD patients suggesting an increase in risk of developing the disease. We hypothesize that concurrent with the beta-secretase activity, cathepsin D may also have amyloidogenic properties through either promoting the formation or reducing the clearance of amyloid. Deposition of PrP amyloid is a characteristic neuropathological finding in vCJD that is only rarely seen in some molecular subtypes of the more common diagnosed forms of sporadic CJD. Continuing studies of vCJD patients must therefore examine PrP amyloid plaque biochemistry and histology to observe the effect of the cathepsin T allele. This may open new avenues of research to allow for a better understanding of the pathogenesis of vCJD.

Identifying genetic factors that increase an individual's risk of developing vCJD may also be beneficial for assessing the relative risk of human-to-human transmission of vCJD, as has been observed via blood transfusion. [3-5,36]

## Competing interests

The author(s) declares that they have no competing interests.

## Availability and requirements

EUROCJD: 

WHO-adopted diagnostic criteria:  

## Authors' contributions

MTB, GGK, and RSGK designed the study. MTB performed the genotyping. PSJ performed statistical analysis. All authors helped draft and approve the final manuscript.

## Pre-publication history

The pre-publication history for this paper can be accessed here:


